# Parasexuality of *Candida* Species

**DOI:** 10.3389/fcimb.2021.796929

**Published:** 2021-12-13

**Authors:** Abhishek Mishra, Anja Forche, Matthew Z. Anderson

**Affiliations:** ^1^ Department of Microbiology, The Ohio State University, Columbus, OH, United States; ^2^ Department of Biology, Bowdoin College, Brunswick, ME, United States; ^3^ Department of Microbial Infection and Immunity, The Ohio State University, Columbus, OH, United States

**Keywords:** *Candida*, parasex, mating, genetic diversity, recombination

## Abstract

While most fungi have the ability to reproduce sexually, multiple independent lineages have lost meiosis and developed parasexual cycles in its place. Emergence of parasexual cycles is particularly prominent in medically relevant fungi from the CUG paraphyletic group of *Candida* species. Since the discovery of parasex in *C. albicans* roughly two decades ago, it has served as the model for *Candida* species. Importantly, parasex in *C. albicans* retains hallmarks of meiosis including genetic recombination and chromosome segregation, making it a potential driver of genetic diversity. Furthermore, key meiotic genes play similar roles in *C. albicans* parasex and highlights parallels between these processes. Yet, the evolutionary role of parasex in *Candida* adaptation and the extent of resulting genotypic and phenotypic diversity remain as key knowledge gaps in this facultative reproductive program. Here, we present our current understanding of parasex, the mechanisms governing its regulation, and its relevance to *Candida* biology.

## Introduction

Species survival depends on the generation of reproductive offspring that are capable of competing in their niche or expanding into new niches. While asexual (clonal) and sexual reproduction are the most common forms of reproduction across kingdoms, evidence for the less known parasexual cycle in nature comes exclusively from fungi and unicellular organisms (Alexopoulos et al., 1996). First described in 1953 by the Italian geneticist Guido Pontecorvo during studies on *Aspergillus nidulans* (Pontecorvo et al., 1953), parasex describes a non-meiotic process of ploidy reduction that produces genetically diverse progeny. Parasex is particularly prevalent in species with no known sexual cycle. While early studies were restricted to filamentous fungi, more recent work has identified parasex in yeast species, with significant relevance to human pathogenic fungi such as *Candida albicans* (*C. albicans*) (Bennett and Johnson, 2003; Forche et al., 2008; Seervai et al., 2013).

*C. albicans* is the most clinically relevant fungal pathogen of humans, owed in large part to being a resident commensal of the oral, dermal, gastrointestinal, and genital niches (Calderone, 2012). Heavy use of steroids or antibiotics, immunosuppression, and autoimmune disorders increase the risk of mucosal and systemic *C. albicans* infections that most commonly result from overgrowth of the patient’s normal flora (Wisplinghoff et al., 2014; Zhai et al., 2020). Since its identification and first description as a species in 1923 (Barnett, 2004), *C. albicans* was long believed to strictly reproduce asexually. In fact, prior to its reclassification into the Saccharomycetes, the *Candida* genus belonged to the Deuteromycetes (imperfect fungi), due to its apparent lack of a sexual cycle (Hibbett et al., 2007).

Early genetic studies of *C. albicans* used parasex-like processes of protoplast fusion and nuclear hybridization between auxotrophic strains to generate hybrid tetraploid strains. The resulting fusant could often maintain a stable karyotype but would undergo random chromosome loss to become aneuploid in some instances (Sarachek et al., 1981; Poulter et al., 1981). As more genetic tools became available, and with the discovery of the mating-competent opaque cell state (Anderson and Soll, 1987) and the mating-type ‘a’ and ‘α’ loci (Hull and Johnson, 1999), the defining features of a non-meiotic parasexual cycle began to take shape in *C. albicans* (Bennett and Johnson, 2003). Here, we review the extant knowledge of the parasexual cycle in *C. albicans*, the underlying molecular mechanisms, parallels to meiosis, and its relevance to natural populations.

## White-to-Opaque Switching as a Pre-Requisite for Mating

The morphological plasticity of *C. albicans* at both the cellular and colony level has long been a focal point of *C. albicans* research, stretching back to its first reported isolation from a thrush patient in 1839 (Knoke and Bernhardt, 2006). The ability to switch between distinct phenotypic states is thought to be central to its ability to thrive in multiple host niches with different pH, metabolite, and oxic profiles (reviewed in (Scaduto and Bennett, 2015). Although more focus has been placed on the yeast-to-hyphal transition because of its tight association with disease, the phenotypic switch between the white and opaque state also regulates several key aspects of *C. albicans* biology.

*C. albicans* is most commonly isolated from clinical infections in the white state, which is characterized by round cells and white dome-shaped colonies on solid medium (‘Candida’ refers to the white robes worn by Senate candidates in the Roman empire and ‘albicans’ means ‘to whiten’ in Latin) (Bennett and Johnson, 2003). In contrast, opaque cells are elongated and club-shaped, forming flat, dull colonies (Rikkerink et al., 1988; Miller and Johnson, 2002; Lohse and Johnson, 2009). Both states are heritable across many generations, with low-frequency transitions between cell states that are governed by a combination of genetic and epigenetic factors (Lohse and Johnson, 2009). Switching to the opaque state canonically requires cells to be hemi- or homozygous for the a or α idiomorph at the Mating-Type Like (*MTL*) locus on Chromosome 5 (Chr5), which has strong similarity to the Mating-Type (MAT) locus in *S. cerevisiae* that determines mating compatibility (Hull and Johnson, 1999; Magee and Magee, 2000). On glucose-containing medium at room temperature and ambient CO_2_, interconversion between the white and opaque states in *MTL* **a** or *MTL*α homozygous cells is rare, occurring only 1 in 10,000 cell divisions (Bergen et al., 1990; Lockhart et al., 2002). However, certain environmental stimuli induce high rates of unidirectional cell state transitions. Conversion to the opaque state dramatically increases in conditions that mimic certain niches in the gut (acidic pH, ≥ 5% CO_2_, and the presence of N-acetylglucosamine (GlcNAc) [reviewed in (Lohse and Johnson, 2009; Noble et al., 2017)]. In contrast, growth on glycolytic carbon sources at 37°C induces conversion to the white state *en masse* (Slutsky et al., 1987; Alkafeef et al., 2018). Interestingly, some heterozygous *MTL* **a**/α isolates can undergo low-frequency white-to-opaque switching under *in vitro* conditions that mimic the gut environment, although the resulting opaque *MTLa*/α state is less stable than its *MTL* homozygous counterparts (Xie et al., 2013).

Consistent with *in vitro* results, *in vivo* passage through the mouse gut induced opaque cell formation in at least one isolate background, WO-1 (Ramirez-Zavala et al., 2008). WO-1 has an extra copy (trisomy) of Chr1, which carries *WOR1*, the master regulator of the opaque cell state (Huang et al., 2006). Similarly, artificial overexpression of *WOR1* in white cells resulted in a competitive advantage in the mouse gut (Pande et al., 2013). Loss of the third copy of *WOR1* decreased recovery of opaque cells from the mouse gut and blocked condition-dependent switching *in vitro* (Huang et al., 2006). Examination of these GUT (‘gastrointestinally-induced transition’) cells showed that they retained *MTL* heterozygosity, were phenotypically stable at higher temperatures (37°C) but lacked ‘pimples’ characteristic for true opaque cells in *C. albicans.* Consequently, this phenotype was considered to be distinct from that of canonical white and opaque cells; its frequency and relevance in natural populations remains unexplored. However, the white state has increased fitness during gut colonization compared to the opaque state in other strain backgrounds and introduced opaque cells will overwhelmingly convert to the white state over time (Pande et al., 2013; Takagi et al., 2019).

White and opaque cell types can be distinguished by distinct physiological, metabolic, transcriptional, and virulence properties. Opaque cells are less metabolically active except when peptides are provided as the sole substrate, which also induce filamentation in opaque cells but not in white cells (Ene et al., 2016). In addition, opaque cells are less susceptible to phagocytosis by macrophages and leukocytes and colonize the heart and spleen more successfully than white cells, potentially resulting in altered pathogenic outcomes (Geiger et al., 2004; Lohse and Johnson, 2008; Sasse et al., 2013; Takagi et al., 2019). The hallmark trait distinguishing white and opaque cells is mating in *C. albicans*. White cells are ‘sterile’ and incapable of mating, whereas opaque cells are mating competent when they are *MTL* hemizygous or homozygous. Mating can occur either between opaque cells of the opposite mating type (i.e., a-α; heterothallic) or between cells of the same mating type (i.e., a-a; homothallic) (Alby et al., 2009; Alby and Bennett, 2011; Guan et al., 2019).

## Mating Requires Specific Molecular Cues That Are Tied to Cell States

**Figure 1:**
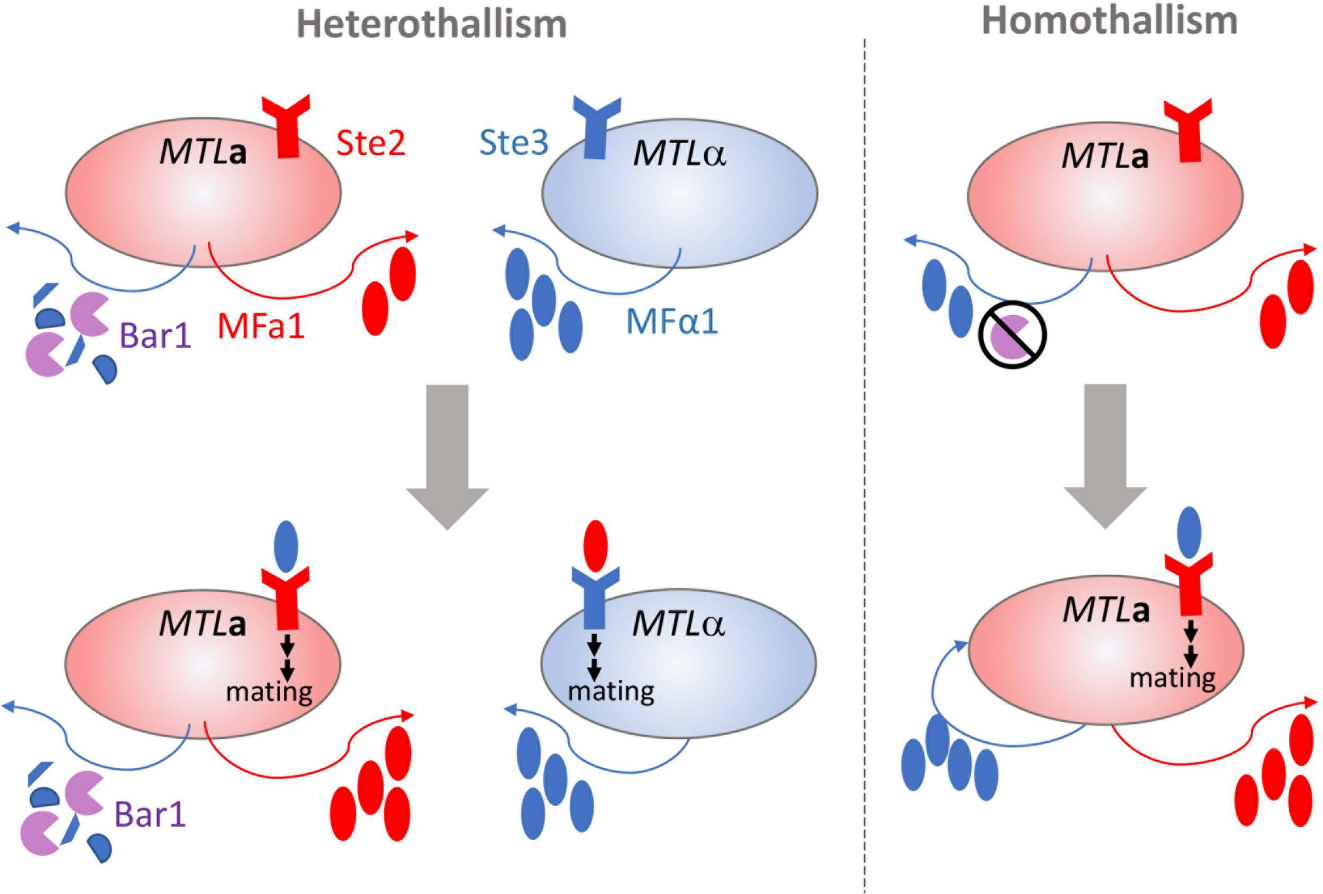
Parasexual mating of opaque cells in *C. albicans*. Opaque cells of the opposite or same mating types (heterothallism and homothallism, respectively) can undergo mating and cell fusion though production and response to pheromone. In heterothallic mating (shown on left), the pheromone receptor is engaged by mating pheromone produced by cells of the opposite mating type to initiate polarized growth and cell fusion (Ste2 receptor by MFα1 or Ste3 receptor by MFa1). Bar1 protease degrades MFα1 produced by opaque *MTL***a** cells and is overcome by the levels of MFα1 produced by opaque *MTL*α cells. In homothallic mating (shown on right), the overproduction of MFα1 or inactivation of Bar1 protease in *MTL***a** leads to autocrine pheromone receptor engagement and a-a cell fusion and mating.

Mating in *C. albicans* requires the molecular sensing of a suitable partner cell followed by a complex series of intracellular events that activate the process of cell-cell fusion. A typical mating cycle in *C. albicans* requires *MTL* **a** and *MTL*α cells to release and sense sex pheromones encoded by the *MFA1* and *MF*α*1* loci, respectively [**Figure 1**, (Bennett et al., 2003; Lockhart et al., 2003; Panwar et al., 2003)]. *MF*α*1* is constitutively expressed in opaque α cells at higher levels than *MFA1* is expressed in opaque a cells. Detection of α-pheromone by opaque a cells induces an increase in *MFA1* and *MF*α*1* levels, fully activating the mating response system in both opaque cell populations. Engagement of the pheromone receptors Ste2 and Ste3 on opaque a and α cells, respectively, initiates polarized growth through activation of the mitogen-activated protein kinase (MAPK) cascade. The polarized cells extend towards the source of the detected pheromone to form mating projections or ‘shmoos’ (Bennett et al., 2003; Lockhart et al., 2003; Yi et al., 2009). This process occurs quickly, typically within ~1.5 h, and halts asexual cell division (Bennett et al., 2003; Lockhart et al., 2003; Yi et al., 2009). Physical contact between opaque cells of opposing mating types results in fusion of the cell cytoplasts and nuclei through a process called karyogamy to produce the zygote, which contains the contents of both parental cells. Mating has most often been observed between diploids to produce tetraploid mating products, but successful mating can also occur between haploid cells (Hull et al., 2000; Bennett and Johnson, 2003; Hickman et al., 2013). Mating products are capable of stably maintaining the resultant ploidy by propagating asexually or can be induced to undergo a disordered ploidy reduction called concerted chromosome loss (CCL) to yield recombinant and karyotypically diverse progeny (Forche et al., 2008; Smith and Hickman, 2020).

Some exceptions exist to the heterothallic mating paradigm for opaque cells. Even though white and opaque cells differentially express ~17% of their transcriptome (Tuch et al., 2010), Scaduto et al. demonstrated that opaque cell fertility was dependent on the activation of only a few genes from the MAPK signaling pathway that are canonically induced by pheromone binding (Scaduto et al., 2017). Ectopic overexpression of the three most transcriptionally silent genes from the MAPK pathway in white cells led to robust pheromone responses and mating at levels comparable to opaque cells. Conversely, disruption of MAPK pathway genes blocks mating in otherwise mating-competent opaque cells even in the presence of pheromones (Magee et al., 2002). This suggests that the MAPK pathway is both necessary and sufficient for parasexual fecundity in *C. albicans*, challenging the tenet that mating competency is exclusively tied to cell state.

**Figure 2:**
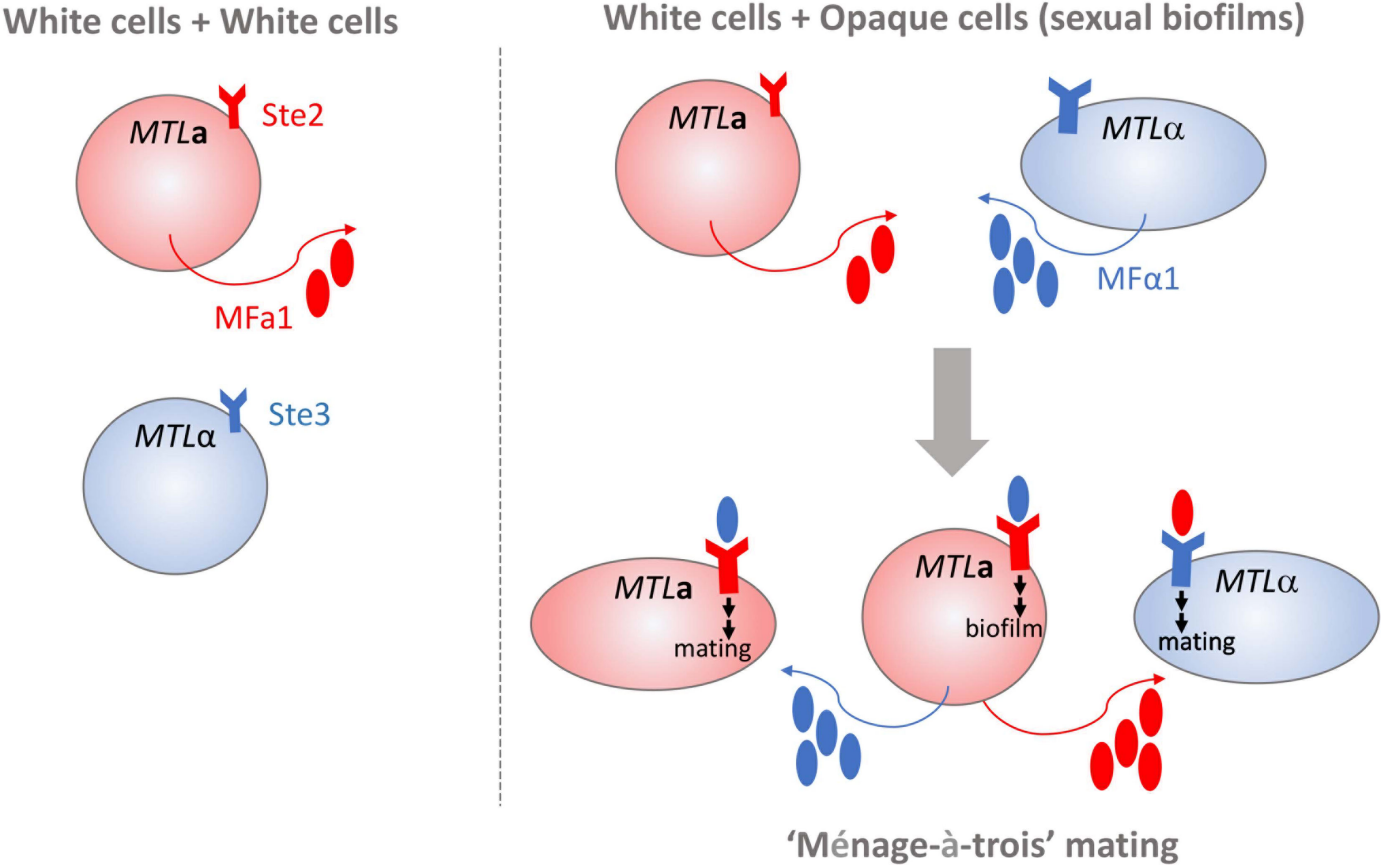
White cells in *C. albicans* mating. White cells (depicted as spherical cells) of opposite mating types do not produce a mating response because of a lack of pheromone production and receptor engagement (shown on left). *MTL***a** white cells exposed to high levels of MFα1 can initiate formation of complex biofilm structures that support opaque cell mating (shown on right). Pheromone-stimulated *MTL***a** white cells can also engage in “ménage-à-trois” mating by supplying MFα1 and MFa1 pheromones to both opaque *MTL***a** and *MTL*α opaque cells (depicted as elongated cells) that induces polarized growth and cell fusion.

Homothallism offers another exception to the heterothallic mating paradigm. Opaque a cells secrete both a- and α-pheromone, which can stimulate paracrine and autocrine signaling responses by engaging the Ste3 receptor on nearby alpha cells or Ste2 on their own cell surfaces [**Figure 1**, (Alby et al., 2009)]. Stimulation of a cells by α-pheromone is typically prevented by the Bar1 protease, which degrades the secreted α-pheromone (Schaefer et al., 2007). However, disruption of *BAR1* or excessive α-pheromone levels can cause *MTL***a** cells to initiate auto MFα1-activated same-sex mating (Alby et al., 2009). Homothallic mating in opaque cells can also be achieved *via* ‘ménage à trois’ mechanisms, in which white cells of the opposite mating type produce pheromones that facilitate mating of opaque cells without mating themselves [**Figure 2**, (Guan et al., 2019)]. Surprisingly, mating pheromone is not necessary for homothallic mating, as opaque *MTL***a** cells can fuse when subjected to environmental stress such as glucose starvation and exposure to oxidative agents (Tao et al., 2014; Guan et al., 2019). Phosphate limitation either through phosphate starvation or mutations in the PHO pathway also increases the mating efficiency of opaque cells, highlighting the myriad environmental conditions that are conducive to initiating the *C. albicans* parasexual cycle without requiring pheromone production or sensing (Zheng et al., 2020).

## Parasex Inside *C. albicans* Biofilms

Pheromone responses also serve to promote the formation of specialized sexual biofilms. Biofilms in *C. albicans* are ordered structures capable of forming on biotic and abiotic surfaces, such as indwelling catheters and other implanted medical devices. Mature biofilms offer protection from immune cell surveillance and antifungal compounds and can serve as reservoirs for repeated infection by actively dispersing fungal cells throughout the patient’s body (Uppuluri et al., 2010; Gulati and Nobile, 2016; Mitchell et al., 2016; Uppuluri et al., 2018). Dissemination can lead to systemic candidiasis in severely immunocompromised patients that is associated with high mortality rates [reviewed in (Nobile and Johnson, 2015)]. The central role of biofilms in disease has prompted new approaches in antifungal drug design that target initiation of biofilm formation and disruption of mature biofilms (Pierce and Lopez-Ribot, 2013; Atriwal et al., 2021).

Whereas conventional biofilms form through a succession of cell adherence, substrate invasion, hyphal formation, and matrix deposition that is dependent on transcriptional regulators (e.g., *BCR1*, *ACE2*) (Fanning et al., 2012; Nobile et al., 2012; Uwamahoro et al., 2012) and cell wall proteins (e.g., Als1, Als3, and Hwp1) (Nobile et al., 2006; Ene and Bennett, 2009; Salgado et al., 2011), sexual biofilm formation initiates in response to the presence of mating pheromone (Lin et al., 2013; Park et al., 2013). Furthermore, conventional and sexual biofilms differ in compactness, antifungal drug resistance, signaling pathways, resilience against host immune responses, and cross-species interactions [reviewed in (Perry et al., 2020)].

Sexual biofilms typically form in an *MTL* homozygous population of *C. albicans* cells. Stochastic transitions to the opaque state initiate secretion of MFa and MFα pheromones that influence cell responses of both opaque and white cells. Daniels et al. (2006) discovered that white cells within pheromone-induced populations respond to pheromones by tightly adhering to a substratum that organizes into a biofilm-like community. The resulting three-dimensional cell matrix facilitates opaque cell mating *via* enhanced chemotropism (Daniels et al., 2006). Mating in sexual biofilms was most efficient with a low percentage of opaque cells (≤ 10%), suggesting that a chemotropism optimum exists to balance formation of a structured environment by white cells with effective migration and mating of opaque cells (Daniels et al., 2006; Park et al., 2013). The immediate presence of white cells may provide additional benefits for mating by promoting homothallism *via* pheromone secretion and protecting opaque cells from the immune response of the host (Perry et al., 2020). Although a fundamental appreciation for the uniqueness of sexual biofilms exists, key questions addressing their *in vivo* relevance and potential to enhance fungal survival and/or pathogenesis remain to be answered.

## Parasexual Ploidy Reduction Promotes Genetic Diversity

Early population genetic studies of *C. albicans* suggested that a meiosis-like mechanism may exist in this species based on the presence of non-clonal genotypes (Graeser et al., 1996; Forche et al., 1999). Yet, the capacity of parasexual mating to generate genotypic diversity through the processes of independent segregation and assortment as seen in meiosis remains poorly characterized. Early drafts of the *C. albicans* genome sequence in the 2000s (Jones et al., 2004; van Het Hoog et al., 2007), genome-wide screens (Magee et al., 2002), and a comparative genomic study (Tzung et al., 2001) provided the groundwork to explore the function of 500 meiosis-related genes from *S. cerevisiae* in *C. albicans*. The *C. albicans* genome retained many mating-related homologs, but some key meiosis regulators were conspicuously absent, such as *IME1* (master regulator of meiosis initiation in *S. cerevisiae*) and *SPO13* (regulator of segregation during meiosis I in *S. cerevisiae*). Subsequent studies identified the presence of several previously overlooked ‘meiosis-specific’ genes in *C. albicans* although the involvement of most homologs in the parasexual cycle remains unstudied [reviewed in (Sherwood and Bennett, 2009; Usher, 2019)].

As parasex does not undergo the traditional organized halving of DNA content through the meiotic cycle, ploidy reduction (depolyploidization) of tetraploid (or diploid) mating products occurs *via* concerted chromosome loss albeit without any clear order for which chromosomes are lost. Ploidy reduction *via* parasex yields highly aneuploid cells with few euploid or near-euploid progeny (Forche et al., 2008; Anderson et al., 2019). Aneuploid progeny preferentially retain extra copies of smaller chromosomes (Chr4-7) that may be less detrimental to the aneuploid cell due to a reduced number of imbalanced genes compared to the larger chromosomes (Forche et al., 2008; Hickman et al., 2015). However, one caveat in most parasexual experiments is that extra copies of Chr1 are selected against because of selection for loss of *GAL1* (located on chromosome 1) with 2-deoxygalactose as a proxy for having undergone ploidy reduction (Gorman et al., 1992; Bennett and Johnson, 2003; Forche et al., 2003; Forche et al., 2008; Thomson et al., 2019). Surprisingly, the imbalanced karyotypes of aneuploid progeny are relatively stable under standard laboratory conditions (Magee and Magee, 1997; Hickman et al., 2015; Anderson et al., 2017). However, serial passaging leads to many aneuploids resolving as diploids, reinforcing this ploidy state as the most stable for *C. albicans* (Hickman et al., 2015). Convergence to diploidy may result from growth advantages of euploid derivatives compared to their aneuploid progenitor in mixed cultures. Indeed, Yang et al. (2021) demonstrated growth defects in rich medium for a panel of strains each trisomic for one of the eight chromosomes. Interestingly, some growth phenotypes were associated with specific chromosome homologs suggesting that inheritance of specific alleles during ploidy reduction can have a larger impact on fitness.

Ploidy reduction *via* CCL can be induced by growth on specific media including glucose-rich ‘pre-sporulation’ (pre-spo) medium, which induces meiosis in *S. cerevisiae* (Bennett and Johnson, 2003). Prior work had shown that induction of CCL on pre-spo medium resulted in the death of most tetraploid, but not diploid cells (Bennett and Johnson, 2003). Recent work by Thomson et al. (2019) elegantly showed that the high glucose content of pre-spo medium selectively pushed the tetraploid cells into a metabolically hyperactive state, leading to higher rates of aerobic and anerobic respiration compared to diploid cells. This in turn led to elevated levels of reactive oxygen species (ROS) as a metabolic byproduct, thereby activating the ROS-responsive transcription factor, *CAP1* (Wang et al., 2006), and causing DNA double-strand breaks (DSBs). These DSBs then served a dual function of destabilizing aneuploid chromosomes and promoting parasexual recombination. This resulted in rapid and random chromosome loss during cell division, giving rise to parasexual progeny with reduced DNA content and potentially recombinant genomes (Thomson et al., 2019).

**Figure 3:**
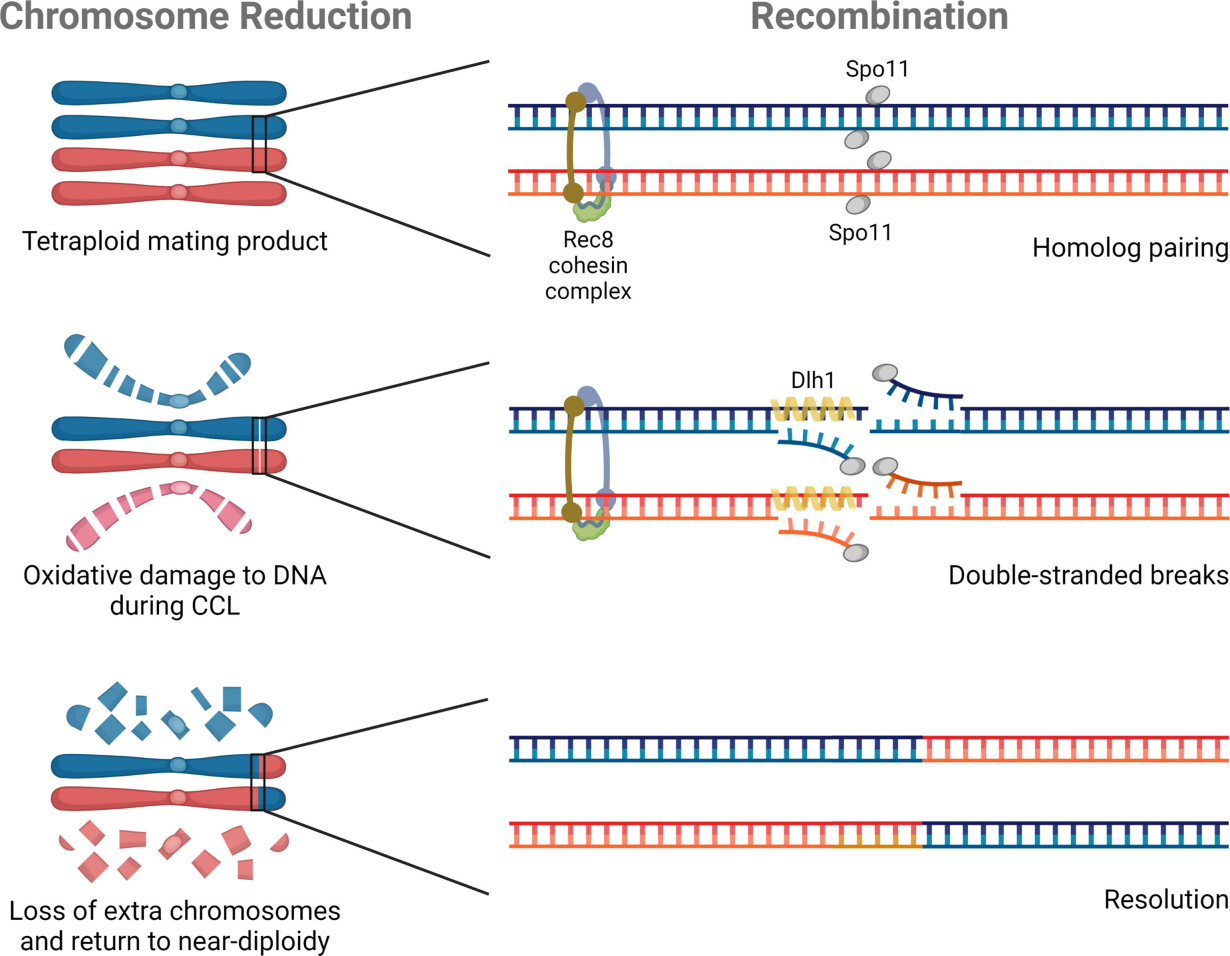
Molecular components of parasexual reproduction. Three genes have experimentally defined roles in *C. albicans* parasexual processes. *SPO11* and *REC8* contribute to ploidy reduction (depicted on the left), and all three genes (*SPO11*, *REC8*, and *DLH1*) are involved in recombination during parasex (shown on the right).

Two meiosis-specific genes have defined roles in *C. albicans* ploidy reduction: *SPO11*, a topoisomerase-that creates double strand breaks, and *REC8*, the kleisin subunit of the cohesin complex (**Figure 3**). Loss of *SPO11* activity increases ploidy reduction whereas disruption of *REC8* decreases chromosome loss (Anderson et al., 2019). These two genes represent the very limited repertoire of ‘meiosis-specific’ genes studied in the context of parasexual ploidy reduction to date.

## Recombination During Parasex

Recombination is a hallmark of meiotic ploidy reduction that is also characteristic of parasex during CCL. The first study of genotypes derived from CCL showed clear evidence of mitotic recombination during parasexual reproduction in *C. albicans* (Forche et al., 2008), with some strains containing multiple recombination events on a single chromosome. Subsequent attempts to quantify the frequency of recombination during parasex demonstrated a greater than 1,000-fold increase in inter-chromosomal recombination than under standard laboratory growth conditions (Anderson et al., 2019). Deletion of *SPO11* abolished all evidence of recombination by comparative genome hybridization and served as the first demonstration that components of the meiotic machinery had been co-opted for parasex (Forche et al., 2008). Surprisingly, *SPO11* was later shown to promote chromosomal stability and mitotic recombination outside of CCL, although the effect was more prominent under conditions that promoted genome instability (Anderson et al., 2019). In this same study, loss of *REC8* similarly decreased parasexual recombination (Anderson et al., 2019). Finally, Ciudad et al. (2020) reported that *DLH1*, a *C. albicans* homolog of the meiosis-specific *S. cerevisiae* factor *DMC1*, could promote strand invasion thereby promoting long-tract loss of heterozygosity (LOH) during recombination (**Figure 3**). However, *DLH1* function was tested outside of parasex through ectopic expression in mitotic cells so the specific role in CCL remains unclear. Described functional roles among diverse proteins in parasex may point to a common central process that is influenced by all these factors or may suggest nonredundant unique roles during recombination that require the individual contributions of Spo11, Rec8, and Dmc1. It remains to be tested if, like mitotic recombination, parasexual recombination might be further facilitated by the presence of defined repetitive elements and cryptic repeat sequences throughout the *C. albicans* genome, namely major repeat sequences (MRS), ribosomal DNA (rDNA) repeats, and telomeric elements (Gusa and Jinks-Robertson, 2019; Todd et al., 2019).

Most parasexual progeny will be inhibited from re-entering the parasexual cycle following CCL by containing 2+ copies of Chr5 and being mating incompatible (*MTL***a**/α). A fraction of the progeny, however, could become *MTL***a** or *MTL*α homozygotes, either during CCL or later through LOH (Popp et al., 2019) and re-enter the mating cycle. Alternatively, haploid progeny could undergo the less commonly observed haploid mating cycle that yields a diploid mating product (Hickman et al., 2013) and/or autodiploidize (i.e., double their DNA content by DNA replication without segregation) to remain mating competent as diploid *MTL* homozygotes. This progeny could then re-enter the parasexual cycle to generate additional mating products. Thus, genetic diversity is produced *via* three distinct mechanisms operating simultaneously during CCL: ploidy reduction, segregation of whole chromosomes, and parasexual recombination.

## Phenotypic Diversity *via* Parasex

The genetic diversity generated by *C. albicans* parasex translates to phenotypic variation in mating products and recombinant progeny. The process of polyploidization during mating (*via* nuclear fusion) serves as a significant driver for phenotypic adaptation by increasing the number of potential karyotypes and genotypes of parasexual progeny as well as changing cell physiology on its own (Harrison et al., 2014; Selmecki et al., 2015). Tetraploid *C. albicans* cells exhibit unique traits compared to genotypically identical diploids such as reduced growth rates and attenuated virulence in a mouse model of systemic disease (Ibrahim et al., 2005; Hickman et al., 2013).

Among parasexual progeny, phenotypic diversity was immediately apparent in the initial studies of parasex. Colonies with altered morphologies and increased or decreased filamentation in Forche et al. (2008) highlighted the implications of parasex towards pathogenesis. A more recent study assessed the fitness of 32 parasexual progeny for multiple traits such as filamentation, virulence, and biofilm formation in comparison to the parental strains (Hirakawa et al., 2017). Parasexual progeny were phenotypically diverse but showed overall reduced fitness compared to their parents in most environments except for increased fluconazole resistance, which was linked to trisomies of Chr3 and Chr6 (Hirakawa et al., 2017).

Chromosome imbalances are known to confer new characteristics to *Candida* (Janbon et al., 1998; Forche et al., 2019), serve as a transient solution to stress in multiple fungal species (Yona et al., 2012; Ford et al., 2015), and are key drivers of phenotypic diversity. Moreover, the tolerance and/or fitness advantages associated with aneuploid chromosomes could increase the probability of beneficial mutations to arise while under selection, negating the need to carry additional chromosomes and their associated fitness costs (Pavelka et al., 2010). In this way, parasex provides a potential means for generating a large reservoir of ploidy-diverse strains for selection to act upon. The potential phenotypic diversity of this progeny pool can be expanded through parasexual mating of genotypically distinct parental strains thereby promoting the exchange of beneficial mutations such as those conferring antifungal drug resistance (Popp et al., 2019).

## Evidence of Parasex in *C. albicans* at the Species Level

A natural question arises from the *in vitro* studies: Has parasex played a role in shaping the *Candida* species phylogeny (Schmid et al., 2016)? In the pre-genomic era, several groups used methods such as enzyme electrophoresis (Pujol et al., 1993), co-dominant molecular markers (Graeser et al., 1996; Forche et al., 1999), and multi-locus enzyme electrophoresis (Arnavielhe et al., 2000) to study the population structure of *C. albicans*. More recent studies used multi-locus sequence typing (MLST) to expand the number of markers and isolates analyzed (Tavanti et al., 2005; Odds et al., 2007). While most studies did not find widespread evidence of recombination, infrequent instances of intra- and inter-locus variation among *C. albicans* isolates in a few studies suggested that genetic exchange through a meiosis-like sexual cycle may be possible (Graeser et al., 1996; Forche et al., 1999; Odds et al., 2007). Ambiguity in the *C. albicans* population structure likely resulted from the study of different populations, different methods, and difficulty in distinguishing parasexual and mitotic recombination in a malleable diploid genome. Difficulty in distinguishing between LOH and parasexual recombination was reinforced in a follow-up a study with 203 isolates (Bougnoux et al., 2008), which concluded that mitotic recombination (leading to LOH events) was widespread among *C. albicans* isolates but there was negligible evidence of parasexual mating.

Analysis of haploid mitochondrial genomes in *C. albicans* offered a simplified context to understand the *C. albicans* population structure without LOH obfuscating inheritance patterns. Contrary to the uniparental transmission of mitochondrial DNA (mtDNA) in most eukaryotes, an early analysis demonstrated low levels of ancient genetic admixture and recombination in the mitochondrial genomes of clinical strains of *C. albicans* and more recent clonal expansions, suggesting that mitochondrial rearrangements may predate *C. albicans* speciation or, alternatively, loss of meiosis (Anderson et al., 2001). Indeed, bi-parental inheritance and recombination in the mitochondrial genomes of *C. albicans* conforms to patterns in other eukaryotes [reviewed in (Barr et al., 2005; White et al., 2008)], which can have some putative evolutionary benefits such as mutational clearance (Neiman and Taylor, 2009). Using the same mitochondrial haplotyping approach, Jacobsen et al. (2008b) reported strong evidence of more recent recombination in a different set of clinical strains. The discordance and inconsistency between the nuclear and mitochondrial genome analyses led to the generally accepted conclusion that, while parasex could be performed in the laboratory, little conclusive evidence existed for it in ‘nature’.

In the post-genomics era, two studies attempted to conclusively address the question of recombination in the *C. albicans* phylogeny. While both studies reported a predominantly clonal population structure, they also revealed robust and recent genetic admixture between different strain clusters (Ropars et al., 2018) and the existence of mosaic genomes shaped by genetic exchange in both nuclear and mitochondrial genomes based on the presence of identical single nucleotide variant (SNV) patterns found in genetically distant strains (Wang et al., 2018). Therefore, the emergent consensus from these phylogenetic analyses suggests that clonal reproduction remains dominant in most *C. albicans* clades with strains having intermittently undergone intra-clade or inter-clade exchange of genetic material, possibly through parasexual recombination.

## Experimental Evidence for Parasex *In Vivo*

Most *C. albicans* strains are isolated as mating incompetent *MTL***a**/α heterozygotes and generally restricted to the white state. This bias towards isolation of the white state, even among *MTL* homozygous strains, may be due to instability of the opaque cell state at the internal human body temperature of 37°C (Alkafeef et al., 2018). Lachke et al. (2003) established that mating can occur on mammalian skin where, at a surface temperature of ~32°C, opaque cells are more stable. Yet, a mixture of *MTL***a** and *MTL*α opaque cells were stable enough to mate upon simultaneous intravenous inoculation in mice (Hull et al., 2000). Furthermore, specific anaerobic growth conditions that mimic niches in the GI tract promote stability of the opaque cell state at internal body temperatures (Dumitru et al., 2007). Indeed, parasexual mating in the GI tract of a murine host was demonstrated by recovering strains that contained both selectable markers from each singly marked parental strain and were uni-nucleate tetraploids by flow cytometry (Dumitru et al., 2007). Despite the evidence for mating *in vivo* using murine models, parasexual progeny resulting from CCL have not been observed in these systems. We are hopeful that future studies will reveal host niches and/or specific environmental conditions that facilitate efficient *in vivo* mating and parasex in *C. albicans*.

## Parasex in Non-*albicans Candida* Species

When did parasex emerge in the *Candida* paraphyletic group? To answer this, most studies have focused on the two species most closely related to *C. albicans*, *C. dubliniensis* and *C. tropicalis*. These three *Candida* species diverged approximately 20 million years ago (MYA) from the *C. orthopsilosis* complex (Moran et al., 2012; McManus and Coleman, 2014). All three species are diploid and share similar traits, including white-to-opaque switching and the ability to mate (Pujol et al., 2004; Porman et al., 2011; Seervai et al., 2013). This suggests that key events in the *C. albicans* parasexual program evolved in the common ancestor before the divergence of these three species.

Consistent with all three *Candida* species having inherited parasex from a common ancestor, they share many characteristics required for mating and CCL. The *C. tropicalis* and *C. dubliniensis* genomes both encode a syntenic *MTL* locus with a similar organization to that of *C. albicans*, where the degree of amino acid identity reflects their phylogenetic relationship (i.e., *C. dubliniensis* being more closely related to *C. albicans* than *C. tropicalis*) (Xie et al., 2012). The *MTL***a** and *MTL*α idiomorphs are present on each homologous chromosome (including all four mating-related genes: a1, a2, α1, and α2) and most isolates are recovered from clinical and environmental settings as *MTL* heterozygotes (Pujol et al., 2004; Xie et al., 2012). Both these species also share features of the parasexual cycle with *C. albicans*: mating between diploid cells, a stable tetraploid zygote, CCL, and the ability for diploid progeny to re-enter the parasexual cycle (Seervai et al., 2013). Similar to *C. albicans*, the population structure of *C. tropicalis* is predominantly clonal with evidence of low levels of recombination among strains that suggests infrequent but detectable levels of sexual exchange among natural isolates (Jacobsen et al., 2008a).

Each species possesses unique attributes of mating and parasex that highlight the divergence in regulatory features of parasex since speciation. For example, the white-to-opaque switching frequency is about an order of magnitude higher in *C. dubliniensis* than in *C. albicans* (Pujol et al., 2004). However, the absence of mating-dependent clumping in *C. dubliniensis* makes intra-species mating less efficient than inter-species mating with *C. albicans* both *in vitro* and *in vivo* (Pujol et al., 2004; Singh-Babak et al., 2021). Inter-species *C. albicans*-*C. dubliniensis* mating products are sterile and unable to either reduce ploidy effectively or re-enter the parasexual cycle. Finally, the grey cell phenotype, found in all three *Candida* species, represents a mating-competent intermediate state along the white-to-opaque transition only in *C. dubliniensis* and *C. tropicalis* (Porman et al., 2011; Anderson et al., 2016; Yue et al., 2016). In *C. albicans*, grey cell formation requires inactivation of the *EFG1* transcription factor, and these cells cannot return to the white state (Liang and Bennett, 2019).

Deeper investigations into *C. tropicalis* parasex has uncovered some unique traits not found in *C. albicans*. Pheromone-assisted a-a homothallic mating occurs independent of the white-to-opaque switch, which is required in *C. albicans* prior to mating (Du et al., 2018). In addition, *C. tropicalis* homothallic and heterothallic tetraploid mating products can undergo mating with diploid cells to generate hexaploid progeny, something never observed in *C. albicans* potentially due to the high instability of >4N cells. Taken together, similarities in parasex between these three species likely reflect their common origin from which each *Candida* species has evolved their own independent parasexual regulatory mechanisms. Retention of parasex over evolutionary time and evidence of recombination in species phylogenies suggests that these mechanisms may be active *in vivo* to provide fitness advantages in the host albeit under yet unidentified conditions.

## Evolutionary Advantages of Parasex

As with all forms of sexual reproduction, parasex is an energetically expensive process but, like meiosis, can promote removal of deleterious mutations in a population and bring together beneficial mutations from genetically distinct lineages. Before evidence of parasexual mating emerged, it was speculated that the *C. albicans* genome contained many detrimental recessive alleles and that the irreversible accumulation of deleterious mutations in an exclusively asexual, diploid organism would lead to progressively declining fitness and eventual extinction (Muller, 1964; Schmid et al., 2016). Even after mating was described in *C. albicans*, the lack of evidence for meiosis-like levels of recombination fueled the hypothesis that rare and cryptic parasex is not enough to confer the benefits of a traditional sexual cycle (Schmid et al., 2004). However, the subsequent discovery of frequent recombination during parasex somewhat disputes this view (Forche et al., 2009; Schmid et al., 2016; Anderson et al., 2019). Furthermore, the genetic and phenotypic diversity generated *via* parasex may improve fitness in colonized or novel host niches. Parasex has the potential to generate genetic diversity at much higher levels than conventional meiosis because of the added karyotypic diversity (Berman and Hadany, 2012), which makes it a useful mechanism for stress adaptation (Yona et al., 2012; Yang et al., 2021). This argument is bolstered by the strong association between exposure to oxidative damage and CCL (Thomson et al., 2019) as well as the phenomenon of stress-induced mating competence in *C. albicans* (Popp et al., 2019). Evidence for niche-specific selection of aneuploidies *in vivo* (Forche et al., 2018; Forche et al., 2019) suggests that parasex may indeed occur in the host but at levels too low to be detected in genetically similar populations without proper tools. In addition, parasex also avoids spore formation that might prove advantageous, especially *in vivo*, because it could limit activation of the host immune system (Heitman, 2010).

One counterargument for the advantages of parasex *in vivo* is the dominance of a clonal population structure in parasexual *Candida* species (Pujol et al., 1993; Boerlin et al., 1996; Forche et al., 1999; Arnavielhe et al., 2000). However, rare sex in otherwise asexually reproducing organisms still has the potential to produce novel genetic variation even from mating between genetically homogenous populations *via* homozygosis of recessive alleles (Masel and Lyttle, 2011).

## Concluding Remarks

Key ancient hybridization events are thought to have played a major role in the evolution of many fungal species and facilitate adaptation to new niches [reviewed in (Steensels et al., 2021)]. Indeed, a hybrid origin was recently proposed for *C. albicans* (Mixão and Gabaldón, 2020) and hybridization events have been detected for non-*albicans Candida* species as well (Pryszcz et al., 2015; Schröder et al., 2016; Mixão et al., 2019; Hovhannisyan et al., 2020; O’Brien et al., 2021). Hybridization often restricts ploidy reduction in sexual and parasexual mating products as has been observed in *C. dubliniensis*-*C. albicans* hybrids, suggesting that species involved in hybridization events were genetically closely related to allow for chromosome loss and continued mating competency. Ploidy reduction of hybridization and parasexual mating products may be aided by the highly plastic genomes of opportunistic fungal pathogens like *C. albicans* that may have evolved to not only allow for the generation of high genetic diversity within populations but also support mixing of otherwise genetically distinct lineages. Both, hybridization events and parasexual mating mark periodic breaks from asexual reproduction at microevolutionary and/or macroevolutionary scales, with potential fitness effects for the population *via* heterosis (hybrid vigor) (Dagilis et al., 2019). The challenge in the future will be to tease apart how and to what extent a variety of evolutionary processes (e.g., hybridization, parasex, aneuploidy, loss of heterozygosity, mitotic recombination, and mutation) have operated independently or in concert to promote the evolution, adaptation, and survival of this species within its mammalian host in general and within specific host niches in particular.

## Author Contributions

Writing - original draft: AM. Writing - review & editing: AM, AF, and MA. All authors contributed to the article and approved the submitted version.

## Funding

This work was also supported by the President’s Postdoctoral Scholars Program fellowship from The Ohio State University to AM. This work was supported by National Institutes of Health grant R01AI148788 and NSF CAREER Award 2046863 to MA. AF was supported by National Institutes of Health grant R15 AI090633.

## Conflict of Interest

The authors declare that the research was conducted in the absence of any commercial or financial relationships that could be construed as a potential conflict of interest.

## Publisher’s Note

All claims expressed in this article are solely those of the authors and do not necessarily represent those of their affiliated organizations, or those of the publisher, the editors and the reviewers. Any product that may be evaluated in this article, or claim that may be made by its manufacturer, is not guaranteed or endorsed by the publisher.
